# Prevention of suicidal behavior with lithium treatment in patients with recurrent mood disorders

**DOI:** 10.1186/s40345-024-00326-x

**Published:** 2024-03-09

**Authors:** Leonardo Tondo, Ross J. Baldessarini

**Affiliations:** 1 Department of Psychiatry, Harvard Medical School, Boston, MA USA; 2 International Consortium for Mood & Psychotic Disorder Research, McLean Hospital, Belmont, MA USA; 3 Lucio Bini Mood Disorder Centers, Centro Lucio Bini, 42 Via Crescenzio, Cagliari and Rome, 00193 Rome, Italy

## Abstract

Suicidal behavior is more prevalent in bipolar disorders than in other psychiatric illnesses. In the last thirty years evidence has emerged to indicate that long-term treatment of bipolar disorder patients with lithium may reduce risk of suicide and attempts, with possibly similar benefits in recurrent major depressive disorder. We review and update selected research literature on effects of lithium treatment in reducing suicidal behavior and consider proposals that higher levels of lithium in drinking water may be associated with lower suicide rates. We summarize results of a growing number of randomized, controlled studies of lithium treatment for suicide prevention including comparisons with placebos or alternative treatments, and comment on the severe challenges of such trials. The basis of a proposed protective effect of lithium against suicidal behaviors remains uncertain but may include protective effects against recurrences of depressive phases of mood disorders, especially with mixed features or agitation, and possibly through beneficial effects on impulsivity, agitation and dysphoric mood.

## Suicidal risks with major mood disorders

Bipolar disorder as well as recurrent major depression (especially if sufficiently severe as to require psychiatric hospitalization) have the highest standardized mortality ratio (SMR) for suicide of any psychiatric disorders, averaging approximately 20-times above the international general population rate of about 15/100,000/year (0.015%/year) (Harris and Barraclough 1997; Tondo et al. 2001, 2003, 2021; Simon and Hales 2012). Bipolar depressive episodes, especially if accompanied by mixed features (APA 2013; Swann et al. 2013; Tondo et al. 2020], are far more likely to be associated with suicidal behavior than mania or hypomania (“[hypo]mania”). In addition, hyperthymic affective temperament seems to exert a selective protective action against suicidal behavior among affective temperament-types (Tondo et al. 2018). Research indicates that suicidal risks overall are high with both types I and II bipolar disorder, with little difference in suicide attempts (Tondo et al. 2007; Novick et al. 2010; Tondo et al. 2016; Fico et al. 2020). However suicides, especially violent acts, are more associated with type-1 disorder and with male sex (Baldessarini et al. 2019; Tondo et al. 2022).

## Suicidal risks and pharmacological treatments

Even though suicidal behavior occurs mainly in depressive states in bipolar disorders, research on the effects of antidepressants on suicidal risk do not provide sufficiently rigorous and consistent information to support either a decrease or an increase of suicide ideation or behavior in mood disorder patients (Maj et al. 2006; Baldessarini et al. 2007; Pacchiarotti et al. 2013; Popovic et al 2015; Braun et al. 2016; Stübner et al. 2018; Hengartner et al. 2021). Increased suicidal risks with antidepressants was firstly reported in the early 1990s (Teicher et al. 1993), leading to widespread discussion in the scientific and lay press, and reviews of evidence supporting and negating this hypothesis (Hammad et al. 2006; Baldessarini et al. 2007). Retrospective analysis of data from controlled trials aimed at supporting licensing of new antidepressants found that suicidal ideation and possibly suicide attempts were increased with antidepressants more than placebos, at least in young adults and juveniles (Hammad et al. 2006; Masi 2022). However, such analyses also found decreased suicidal risks in older adults, with an overall outcome of no difference without separation by age-groups (Hammad et al. 2006; Barbui et al. 2009; Kahn et al. 2011; Wise 2016). For major depressive disorder, studies have found both increases and decreases in suicidal ideation in patients treated with antidepressants versus a placebo (Acharya et al. 2006; Yerevanian and Choi 2013), or no difference for suicide risk but a significant increase of suicide attempts in antidepressant-treated participants (Braun et al. 2016). Use of antidepressants in bipolar depression is not recommended, particularly with type I bipolar disorder, to avoid inducing potentially dangerous manic episodes, whereas their use for bipolar II depression seems safer but remains unsettled (Pacchiarotti et al. 2013; Baldessarini et al. 2020).

Among innovative treatments for depression, evidence is growing that the glutamate NMDA-receptor antagonist ketamine and its *S*-enantiomer (esketamine) can exert rapid, short-term reduction of symptoms of depression (Nikolin et al. 2023). Such effects have been found in otherwise treatment-resistant unipolar depression and in bipolar depression, often with at least temporary reduction of suicidal ideation but with unknown effects on suicidal behavior (Baldessarini et al. 2020; Zisook et al. 2023).

Few studies have tested for an association between antipsychotic treatment and suicidal risk in mood-disorder patients. At least one randomized trial of clozapine versus olanzapine supports efficacy of clozapine against suicidal or suicide-associated behavior in schizophrenia patients, though without reduction in risk of rare suicides (Meltzer et al. 2003; Hennen and Baldessarini 2005). However, whether its antisuicidal actions in schizophrenia extend also to bipolar disorder remains to be tested, although there is some evidence of clinical effectiveness of clozapine in bipolar disorder, especially for patients who have not responded satisfactorily to other treatments (Ifteni et al. 2014; Li et al. 2015) or have psychotic features (Ciapparelli et al. 2000). Other modern antipsychotic drugs have substantial and growing evidence for efficacy and safety in the treatment of bipolar depression (Fornaro et al. 2016; Post 2016) but they require testing as a potential option to reduce suicidal risk in bipolar disorder patients.

## Early evidence concerning lithium treatment and suicidal risk

A first report on the possible prevention of suicidal behavior with lithium is attributed to Barraclough (1972). The study hypothesized that treatment with lithium would have avoided suicide in about 20% of suicides by preventing depressive recurrences alone. A first review on the association between long-term lithium treatment and lower rates of suicides and attempts based on 28 clinical comparisons of over 17,000 participants reported an 8.60-fold lower rate during treatment with than without lithium (Tondo et al. 1997). A later review of 31 clinical reports (including 5 randomized, controlled trials) found a meta-analytically pooled, 4.91-fold lower risk of suicides and attempts in patients with recurrent major mood disorders (Baldessarini et al. 2006).

In addition to the early favorable observations by Barraclough (1972), a double-blind study (Prien et al. 1974) of 249 bipolar and 78 unipolar depressed patients, found two suicides associated with placebo-treatment (1.28%/year) and none with lithium treatment during 24 months of follow-up. Kay and Petterson (1977) also found no suicides in 123 patients followed briefly in a specialized lithium clinic. Poole et al. (1978), in a comparison of five years before versus during lithium treatment, found half as many suicide attempts during treatment of 100 patients with various mood disorders.

Another early study by Hanus and Zalpetálek (1984) found a six-fold lower annual rate of suicide attempts among 95 patients with various recurrent major affective disorders, based on comparing rates before versus during an average of 5.1 years of lithium treatment. Lepkifker et al. (1985) compared the rate of suicide before versus during lithium maintenance treatment for an average of 8.3 years in 33 unipolar depressive disorder patients: 21.2% had attempted suicide before treatment (2.55%/year) compared to none during lithium treatment.

Coppen and his colleagues (1991) also found no suicides in 103 major affective-disorder patients attending a lithium clinic for 11 years (< 0.088%/year). A later extension of this study identified one suicide in a total of 1519 patient-years (0.066%/year) of lithium maintenance treatment for major affective disorder Coppen and Farmer (1998). Moreover, a 13.8-fold higher rate (0.91%/year) was found in 27 untreated unipolar depressed patients, again suggesting a possible protective action of lithium in major depressive disorder (Coppen et al. 1998).

Müller-Oerlinghausen and collaborators (1992) designed an early case–control study of suicidal risk in 68 patients with various major affective disorder and at least one suicide attempt, before, during an average of 8.0 years of lithium treatment, and following its discontinuation. They found six suicides or attempts during treatment (1.10%/year) but 11 after lithium discontinuation (2.02%/year). Later, Nilsson (1999) found a 4.8-times (CI 1.10–12.6; *p* = 0.02) higher rate of suicides in patients who discontinued lithium compared to when they had been receiving lithium treatment.

We studied the suicidal behaviors of 360 type I or II bipolar disorder patients before, during, and following discontinuation of long-term lithium monotherapy, and found that rates of suicide and life-threatening attempts were 6.4-fold lower during lithium-treatment than either before or long after treatment was discontinued (Tondo et al. 1998). Notably, however, the risk of suicidal acts increased by 20-fold within several months after discontinuing lithium maintenance treatment, and later fell back to the same level encountered before lithium treatment had started. This early suicidal risk following discontinuation of long-term treatment with lithium was twice-higher following abrupt or rapid versus gradual discontinuation of lithium (< 14 vs. ≥ 14 days). These studies were included in a meta-analysis (Tondo et al. 2001) in which the pooled rate of suicides and attempts without lithium was 1.02%/year, and nearly nine-times lower during long-term lithium-treatment, with a computed risk-ratio (without versus with lithium-treatment) of 8.85 (95% CI 4.14–19.1; *p* < 0.0001).

## Further studies of lithium treatment and suicidal risk

In line with earlier reports, our later meta-analysis (Tondo et al. 2003) of 28 studies involving 823 suicides among 21,484 bipolar disorder patients at risk for an average of 9.93 years, found a pooled, weighted mean annual incidence of suicide without lithium of 0.39%/year (390/100,000/year, or per 100 k PEY)—more than 20-times higher than the international suicide rate in the general population, of approximately 0.015%/year (Miola et al. 2023).

The very high risk of suicide among bipolar disorder patients also was supported by our subsequent assessment of the medical records of nearly 3000 outpatients diagnosed with DSM-IV major mood disorders (Tondo et al. 2007). In that study, the risk of suicide among bipolar disorder patients was similar in bipolar disorder types I and II, and both rates were higher than among patients diagnosed with unipolar major depressive disorder, overall, but similar among patients of all three types who ever required psychiatric hospitalization. The annual risk of *suicide* among 843 bipolar I and II disorder patients in Sardinia was 150/100 k PEY, or more than 15-times higher than in the Italian general population, and three-times greater than among 1983 patients diagnosed with recurrent major depressive disorder, most of whom were outpatients. The annual rate of *suicide attempts* was 1.26% among bipolar disorder patients versus 0.48% in unipolar depressive cases.

We proposed the ratio of attempts/suicides (A/S) as an *index of lethality*. It was twice greater during treatment with lithium, suggesting *reduced lethality* of suicidal behavior with this treatment, or fewer fatalities per attempt within a group of patients (Baldessarini et al. 2006). The *index of lethality* of suicidal behavior was 8.6 in bipolar disorder patients, and 9.6 in unipolar depression patients, compared to an estimated A/S risk-ratio close to 30 in the general population (Kessler et al. 2005; Miola et al. 2023). These observations indicated relatively high lethality of suicide attempts among mood disorder patients generally, and particularly among bipolar disorder patients—presumably reflecting both intent to die and employment of relatively lethal methods.

In addition, in a meta-analysis of eight studies of patients with unipolar recurrent major depression, we found that long-term lithium treatment was again associated with a substantial reduction of risk of suicides and attempts (by approximately 76%) among patients treated with lithium compared to other alternatives, mainly anticonvulsants (Guzzetta et al. 2007). Three more recent reviews (Cipriani et al. 2013; Turecki and Brent 2016; Abou-Saleh et al. 2017) added further support for a beneficial effect of lithium on suicidal behavior in major depressive disorder.

Our 2006 meta-analysis of 31 studies included five trials randomized to treatment with lithium versus placebo or alternative active medicines (Baldessarini et al. 2006). In these studies, suicidal behavior was ascertained along with other adverse outcomes in an informal and incidental manner. They contrast to the rare InterSEpT trial of clozapine versus olanzapine in suicidal schizophrenia patients, explicitly designed to detect suicide-related behavior as the principal outcome measure (Meltzer et al. 2003). The meta-analysis of five controlled trials involving lithium treatment provided data on suicidal behavior in mood-disorder patients treated clinically, long-term, with lithium or a comparator or a placebo involving more than 110,000 person-exposure-years (PEY) of risk (Baldessarini et al. 2006).

In these studies, crude rates of suicidal acts averaged 0.56%/year (or per 100 person-years) with lithium and 2.64%/year without lithium, indicating a major overall estimate of about five-fold lower suicidal risk with lithium treatment. Apparent reductions were similar for both suicides and attempts, and among purely bipolar disorder patients and samples of persons with various recurrent major mood disorders. In the 31 available studies, we found an overall lower suicidal risk by approximately 80% (risk ratio = 4.91, CI 3.82–6.31; *p* < 0.0001). Results remained highly significant when only the five randomized, controlled trials (RCTs) were considered (RR = 1.76, CI 1.65–1.88; *p* = 0.001). When we further extended this analysis in 2008 to include 34 studies, we found similar reductions of risk and estimated a number-needed-to-treat (NNT) of 23 (CI 21–25) (Baldessarini and Tondo 2008). We reported similar results for 12 randomized trials in ten reports including both bipolar and major depressive disorder patients (Tondo and Baldessarini 2018). The preceding findings are summarized in Table 1.

**Table 1:** Meta-analytic findings of studies of lithium versus risk of suicides or attempts: summary

Conditions	Studies (n)	Risk Ratio (RR) [95% CI]	*p*-value
All studies	35	3.77 [2.72–5.24]	< 0.0001
*Outcomes*			
Suicides	15	4.03 [2.37–6.85]	< 0.0001
Attempts	8	3.73 [1.50–9.33]	< 0.005
Both	12	3.66 [2.28–5.87]	< 0.0001
*Diagnoses*			
Bipolar	14	3.84 [2.34–6.30]	< 0.0001
Major affective	21	3.72 [2.40–5.77]	< 0.0001
Major depression	8	4.69 [1.66–13.3]	< 0.0001
*Trial types*			
Open	29	3.95 [2.79–5.58]	< 0.0001
Randomized, placebo-controlled	6	1.91 [0.89–4.08]	0.096
*Comparator type*			
vs. placebo or no lithium	27	4.22 [2.91–6.10]	< 0.0001
vs. anticonvulsants	8	2.42 [1.15–5.10]	0.02

Analyses based on random-effects modeling, with stratification. Data from Baldessarini et al. (2006), Guzzetta et al. (2007), Collins and McFarland (2008), Lauterbach et al. (2008), Smith et al. (2009), and Oquendo et al. (2011). RR is for differences between lithium vs. comparators (values > 1 favor lithium)

Several more recent studies provide additional data. One is a rare randomized, controlled study (Lauterbach et al. 2008) which found a substantial but statistically nonsignificant difference in rates of suicidal acts between major affective disorder patients treated for 12 months with lithium versus placebo (adjusted hazard ratio [HR]: 0.52 [CI 0.18–1.43], not significant). However, post-hoc analysis revealed that all of the three suicides observed occurred in the placebo-treated group. In another randomized comparison of lithium versus placebo added to the antidepressant citalopram for 4 weeks to treat bipolar disorder patients, suicidal behavior and ideation were rated with the Montgomery-Åsberg Depression Rating Scale and the Sheehan-Suicidality Tracking Scale, Beck Suicide Scale (BSS) and Beck Hopelessness Scale (BHS) (Khan et al. 2011). No suicides or attempts occurred in either group, making the data inconclusive. However, all scores from the rating scales decreased significantly more with the combination of lithium with citalopram than with placebo. In addition, a study based on retrospective data from a sample of 161 bipolar disorder patients found that rates of nonlethal suicide-related events were lower during long-term lithium treatment than with other mood-stabilizers given alone or with various types of antipsychotic drugs (Koek et al. 2012).

The association of reduced suicidal risk with long-term treatment with lithium in bipolar disorder or recurrent major affective disorder patients is supported consistently by nearly three-dozen original studies, meta-analyses and reviews (Tondo et al. 2001, 2003; Angst et al. 2005; Cipriani et al. 2005; Kessing et al. 2005; Müller-Oerlinghausen et al. 2013; Riblet et al. 2022; Wilkinson et al. 2022). Also, a large study based on more than 30,000 subjects (age > 15) with a total exposure of about 267,000 person-years confirmed a lower rate of suicide and self-harm associated with lithium treatment (Fitzgerald et al. 2022). Another recent study found reduced risk of psychiatric hospitalization and of suicidal behavior among both bipolar and major depressive disorder patients for whom lithium was added to their treatment regimens (Pompili et al. 2023).

Beneficial effects of lithium in reducing all-cause mortality have been reported since the early 1990s (Müller-Oerlinghausen et al. 1991; Müller-Oerlinghausen et al. 1992a, b; Toffol et al. 2015; Abou-Saleh et al. 2017). In particular, cardiovascular mortality was reduced during lithium treatment (Ahrens et al. 1995). In addition, mortality increased after discontinuation of lithium treatment (Müller-Oerlinghausen et al. 1996). Reduction of mortality or morbidity associated with suicidal behaviors is especially remarkable given the potentially lethal toxicity of lithium in acute overdoses. In this regard, analysis of outcomes of ingestions of potentially toxic substances indicates that mortality from overdoses of lithium (13.2 deaths per 10,000 overdoses) was greater than with modern antipsychotics (5.80/10,000) but lower than with acetaminophen (25.8/10,000) or tricyclic antidepressants (40.7/10,000) (Nelson and Spyker 2017). Lethal outcomes may be limited by protection from vomiting as well as by timely hemodialysis (Watson et al. 2003; Baldessarini 2013). Despite lithium’s potential lethality, suicide attempts with lithium during long-term treatment appear to be uncommon (Waddington and McKensie 1994). None was found among more than 700 patients with major mood disorders treated with lithium for several years at the Lucio Bini Mood Disorders Center in Sardinia (Tondo 2023). It is possible that suicide attempts using this agent are limited by antisuicidal effects or modulation of impulsivity and aggressive behavior.

**Fig. 1:**
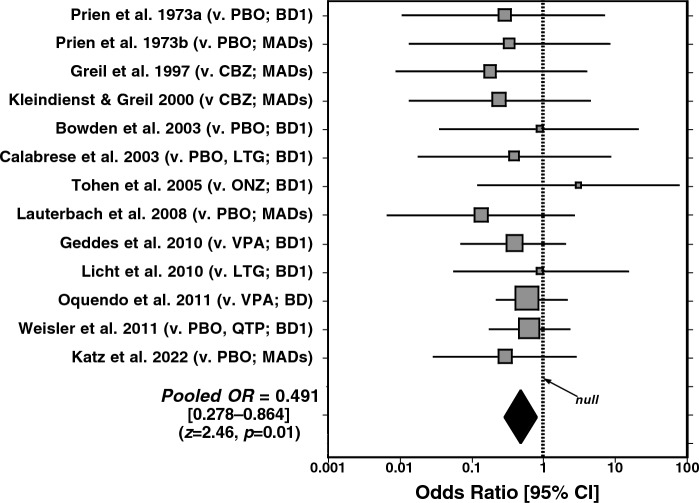
Forest plot of meta-analysis of 13 randomized, controlled trials of rates of suicidal acts in patients with type I bipolar disorder (BD1), any bipolar disorder (BD), or mixed major mood disorders (MADs) treated with lithium versus placebo or alternative mood-stabilizing or antipsychotic agents (carbamazepine [CBZ], lamotrigine [LTG], olanzapine [ONZ], quetiapine [QTP], or valproate [VPA]). The pooled (black diamond) Odds Ratio (OR) is 0.491 [CI: 0.278–0.864], favoring lithium (z-score = 2.46, p = 0.01)

For the present report, we gathered data from a total of 13 randomized, controlled trials (RCTs) of lithium involving 3836 subjects given lithium (n = 1498) versus either placebo, a mood-stabilizing anticonvulsant, or a modern antipsychotic (n = 2338 participants; Table 2). All subjects were diagnosed with either bipolar disorder or a variety of recurrent major affective disorders. Additional studies involving unipolar major depression were excluded, as were uninformative trials with no suicidal events with either treatment-type. The crude pooled rate of suicides and attempts with lithium was 14/1498, or 0.935% [0.512–1.56] versus 36/2338, or 1.54% [1.08–2.13] with alternative treatments. Corrected for mean exposure time of 1.73 years in both groups, the rate per 100 k PEY averaged 540 with lithium versus 810 with placebo or other treatments, indicating a 1.65-fold lower risk of suicidal behaviors with lithium. Data from the same 13 RCTs was also subjected to meta-analysis (Fig. 1) and yielded an overall Odds Ratio of 0.491 [0.278–0.864] favoring lithium (*z*-score = 2.46, *p* = 0.01). It is important to point out again that suicidal behavior was not an explicit, a priori, outcome measure, but was instead noted among adverse events.

**Table 2:** Suicidal acts in 13 randomized controlled trials of lithium

Trial	Diagnosis	Acts/subjects/years		Other treatment	RR (n/N/years)
		Lithium	Comparator		
Prien et al. 1973a	MAD	0/45/2.0	1/39/2.0	Pbo	> 1283
Prien et al. 1973b	BD	0/101/2.0	1/104/2.0	Pbo	> 481
Greil et al. 1997	BD	0/74/2.5	2/70/2.5	CBZ	> 1143
Kleindienst and Greil 2000	MAD	0/86/2.5	6/285/2.5	CBZ	> 842
Bowden et al. 2003	BD	0/46/1.5	1/129/1.5	LTG/Pbo	> 310
Calabrese et al. 2003	BD	0/121/1.5	2/242/1.5	LTG	?551
Tohen et al. 2005	BD	1/214/1.0	0/217/1.0	ONZ	< 1.0
Lauterbach et al. 2008	MAD	0/84/1.0	3/83/1.0	Pbo	> 3614
Geddes et al. 2010	BD	2/110/2.0	5/110/2.0	VPA	2.50
Licht et al. 2010	BD	1/78/1.0	1/77/2.5	LTG	1.01
Oquendo et al. 2011	BD	6/49/2.5	8/49/2.5	VPA	3.40
Weisler et al. 2011	BD	3/346/2.0	11/808/2.0	QTP/Pbo	1.96
Katz et al. 2022	BD	1/144/1.0	3/125/1.0	Pbo	3.46
Totals/mean years	–	14/1498/1.73	36/2338/1.73	–	–
Rate (%) [95% CI]	–	0.935 [0.512–1.56]	1.54 [1.08–2.13]	–	–
Rate/100 k PEY	–	540	810	–	1.65

Outcome is suicide or attempt in RCTs vs. placebo or an alternative mood-stabilizer with at least one suicidal act in one of the two arms as a numerator (n). *BD* bipolar disorder, *CBZ* carbamazepine, *LTG* lamotrigine, *MAD*, major affective disorders, *ONZ*, olanzapine, *Pbo* placebo, *PEY* person-exposure-years, *QTP* quetiapine, RR rate ratio (based on rates as n/N/years as per 100 k PEY); *VPA* valproate. Data with references are from recent reviews: Baldessarini et al. (2006); Cipriani et al. (2013); Lewitzka et al. (2015); Del Matto et al. (2020). Based on meta-analysis (Fig. 1), the pooled Odds Ratio (OR with 95% CI) was 0.491 [CI: 0.278–0.864] (*z*-score = 2.46, *p* = 0.01)

A very recent report found a beneficial effect against mortality with lithium and secondarily with sodium valproate is a Taiwanese nationwide cohort survey (Chen et al. 2023). Based on nearly 26,000 participants with bipolar disorder who had been hospitalized, the study considered effects of the use of mood-stabilizers in all-cause and natural mortality as well as suicides. Suicide accounted for 19.0% of the mortality in the cohort with an SMR of 26.0-fold. Lithium was associated with highly significantly lower risks of all-cause mortality, suicide, and natural mortality—even lower than with sodium valproate, lamotrigine, or carbamazepine.

## Challenges and controversies in research on suicide prevention

Consideration of lithium as a potential antisuicidal treatment has not been without serious ethical and technical challenges and some controversy. A recent illustrative example involves a randomized trial interrupted for futility after several months as suicide-related events in 519 subjects from a US Veterans Administration (VA) population remained similar in lithium and placebo-treated cases (Katz et al. 2022). Despite being reported in a highly influential journal, the study results should be taken with great caution and considered inconclusive rather than as compelling evidence of a lack of benefit of lithium. Problems encountered included: (a) serum lithium levels lower than are employed in standard clinical practice and additional uncontrolled treatments were allowed; (b) lithium treatment was brief and probably insufficient to reveal possible beneficial effects; (c) patients at relatively high suicidal risk, with multiple previous suicidal acts, were excluded; and (d) diagnoses were highly heterogeneous, including unipolar depression as well as co-occurring substance abuse and other disorders (Manchia et al. 2022).

Another recent negative review of evidence regarding lithium treatment and suicidal risks by Nabi et al. (2022) considered 12 RCTs with 2578 participants and found no difference between suicide rates during treatment with lithium or placebo. This review stirred a reaction by the International Group for The Study of Lithium Treated Patients (IGSLI) (Bschor et al. 2022) questioning the validity of the conclusions. Criticisms noted included: (a) exclusion of trials carried out before the year 2000); (b) selective inclusion of some but not all studies from an earlier meta-analysis (Cipriani et al. 2005); (c) inclusion of studies of lithium versus placebo only and excluding comparisons to active comparators; (d) inclusion of studies without information on suicide but assuming that there were no suicides.

In general, gathering of evidence to test the hypothesis that lithium or other treatments may exert suicide-sparing effects is very difficult. Evidence from randomized, controlled trials (RCTs) is greatly to be desired. However, the design and conduct of such trials is highly challenging both ethically, with death as a potential outcome, and technically. Ethical considerations often compel designs that compare a research treatment of specific interest to an alternative treatment that might also have antisuicidal effects, as well as especially close monitoring and protection of study participants as in the InterSePT trial (Meltzer et al. 2003). Technical challenges include recruitment of adequate numbers of willing subjects at substantial or even high risk of suicidal behavior, and their retention for sufficient time as to support an outcome of interest. Owing to the rarity and danger of suicides and attempts, it is tempting to employ surrogate outcomes, such as threats of suicide or the need for clinical intervention to avoid suicide, which may or may not be reliable substitutes for actual suicidal behavior. Much of the available evidence from RCTs involving lithium, as summarized in Table 2, has yielded tiny numerators for suicidal rates, often as low as zero or one or two, making for unreliable statistical analyses and yielding largely suggestive evidence.

Moreover, as noted, the recording of suicidal behavioral events has typically involved reports of incidentally reported adverse events rather than suicidal behavior as an explicit outcome measure, with risks of inaccurate reporting. Such designs usually are considered inadequate for regulatory support of a therapeutic indication for antisuicidal effect. It is therefore not surprising that the InterSePT trial of clozapine versus olanzapine is a rare and compelling trial of a medicinal treatment to reduce risk of suicide, and even it relied considerably on surrogate measures and actually encountered a few (nonsignificantly) more suicides and all-cause deaths with clozapine than with the comparator olanzapine [Meltzer et al. 2003]. Given these realities, conclusions and clinical policies regarding possible antisuicidal effects of experimental treatments remain based on alternative sources of evidence to RCTs with suicidal behavior as the explicit outcome of interest.

## Lithium in drinking water

A provocative finding has been of lower rates of suicide and other violent behaviors in some studies of populations exposed to drinking water with relatively high concentrations of lithium as a naturally occurring mineral (Brown et al. 2018; Barjasteh-Askari et al. 2020). Of note, such concentrations of lithium involve daily intakes far lower than those required for treatment of bipolar disorder (Baldessarini 2013).

An early study on this topic found lower rates of hospital admissions and homicide in Texas counties with higher lithium levels in drinking water (Dawson et al. 1972). A reduction of rates of suicidal or other aggressive behaviors was found in other studies at lithium levels of 0.40–31.5 µEq/L (2.78–219 µg/L) in drinking water (Blüml et al. 2013), averaging 11.3 µg/L (78.4 µg/L) (Kapusta et al. 2011). Similar findings were reported in a Japanese study that involved lithium concentrations of 0.7 to 59 µg/L (0.1–8.5 µEq/L) (Ohgami et al. 2009), in another large nationwide Japanese study (Kugimiya et al. 2021), and in a recent Hungarian study (Iszak et al. 2022) with lithium levels averaging 14.3 µg/L (range, 0.71–303 µg/L). Another Texas study found a significantly higher local incidence of suicide, homicide, rape and other criminal behaviors in 27 Texan counties with very low or undetectable lithium in drinking water compared to counties with lithium concentrations of 70–170 µg/L (Schrauzer and Shrestha 1990).

The significance of these provocative findings is far from clear, especially since the lithium exposures were very low by comparison to clinical doses of 300–1500 mg/day of lithium carbonate (Li_2_CO_3_; 8–40 mEq of lithium ion) to yield daily minimum serum concentrations of 0.6–0.9 mEq/L for treating bipolar disorders (Baldessarini 2013). Although only a minority of studies have found beneficial effects of lithium in drinking water in relation to suicidal behavior, some investigators have been sufficiently convinced by available research findings as to propose addition of lithium to drinking water (Memon et al. 2020; Araya et al. 2022; Rihmer and Dome 2023).

Findings from 25 reports on this topic are summarized in (Table 3). They are inconsistent, in that only 8/25 (32.0%) reports supported a lower risk of local suicide rate with higher concentrations of lithium in the corresponding supply of drinking water at levels that varied widely across regions (0.10 to 80 µg/L). Of note, 6/25 reports (24.0%) found no evidence of an inverse association of water concentrations of lithium and suicide rates and another 11/25 studies (44.0%) produced inconsistent evidence of a favorable association between suicide rates and lithium levels in local water supplies. The inconsistent findings included benefits mainly among men (9/25 reports, 36.0%) or among women (2/25, 8.00%), only among those living at relatively low altitudes (2/25, 8.00%), or with risks limited to specific periods of time (1/25, 4.00%).

A further reason for skepticism about the possible psychiatric value of lithium in drinking water is recent evidence of its association with increased risk of autism spectrum disorders of the offspring of mothers exposed to relatively high levels of lithium (Liew et al. 2023). Nevertheless, some investigators have suggested that lithium be added to local water supplies for possible benefits versus suicide or other aspects of health such as proposed but uncertain reduction of risk of dementia (Brown et al. 2018; Ishii et al. 2021; Rihmer and Dome 2023), much as has been done with fluoride to protect against dental caries (Iheozor-Ejiofor et al. 2015).

Another approach to testing for relationships of environmentally available lithium and suicidal risk is to assay lithium concentrations in body-fluids. A recent, small, pilot study assayed low, naturally occurring concentrations of lithium in aqueous fluid taken from the eye postmortem. It found ophthalmic lithium levels among suicides (0.45 µg/L) to be half that of others dying of natural causes (0.90 µg/L) (Ando et al. 2022).

## Other mood-stabilizing treatments

There is little research that directly compares suicidal risks during treatment with mood stabilizing agents other than lithium, and available findings usually have favored lithium (Thies-Flechtner et al. 1996; Goodwin et al. 2003; Baldessarini and Tondo 2009; Koek et al. 2012). At least two studies have found nearly three-fold average lower risk of suicidal behavior with lithium than with either carbamazepine or valproate given to bipolar or schizoaffective disorder patients (Thies-Flechtner et al. 1996; Goodwin et al. 2003). Lithium also was found more effective than valproate especially in patients older than 42 years (Dervic et al. 2023). Although anticonvulsants may have some beneficial effects on suicidal behavior (Yerevanian et al. 2007), our meta-analysis of protective effects against suicidal behavior of lithium versus several proved or putative mood-stabilizing anticonvulsants (including carbamazepine, lamotrigine, and valproate) regarding suicidal acts found lithium to be significantly superior (Baldessarini and Tondo 2009). That meta-analysis was based on six studies with more than 30,000 patients, but they were treated longer with lithium (31 months) than with an anticonvulsant (19 months). Half of the trials involved randomized assignment to treatments. The observed rate of suicidal acts averaged 0.3%/year during treatment with lithium versus 0.9%/year with anticonvulsants, to yield meta-analytically pooled risk ratio of 2.86 (CI 2.29–3.57; *p* < 0.0001), or nearly three-fold superiority favoring lithium over the few anticonvulsants that have been tested in this way.

A Danish national pharmacoepidemiological cohort study of over 16,600 persons sampled for 6 years found significantly fewer suicides among patients filling prescriptions for lithium or valproate in addition to an antipsychotic drug, compared to antipsychotic treatment alone (Smith et al. 2009). Another controlled and randomized, but under-powered trial found no difference in rates of suicidal behavior among 35 potentially suicidal bipolar disorder patients randomized to lithium or valproate (without or without other uncontrolled treatments) for up to 30 months (Oquendo et al. 2011). Finally, a study from the US Veterans Administration of more than 1000 patients found reduced incidence rates for attempted suicide ranking: lithium plus divalproex < divalproex alone < lithium alone (Ahearn et al. 2013). It remains unclear whether these studies support the hypothesis that both valproate and lithium exert antisuicidal effects or are simply inconclusive.

## Comments

The findings reviewed here were based on data from reports and meta-analyses of studies reporting on associations of suicides and attempts with versus without long-term lithium treatment, including some randomized, placebo- or alternative drug-controlled, trials (Table 2). With few exceptions, usually involving inconclusive study-designs, the findings indicate lower rates of suicidal behavior with long-term lithium treatment in bipolar disorder patients, and suggestive or inconclusive evidence concerning comparisons of lithium versus anticonvulsant or antipsychotic drugs commonly used in the treatment of bipolar disorder (Table 1). Such effects, though particularly strong among bipolar disorder patients, also were found among samples involving various recurrent major affective disorders, including unipolar recurrent major depression .

**Table 3:** **Studies of lithium in drinking water versus **suicide rate

Measure	Outcome
Reported studies (1972–2022)	25
*Countries* (%)	
Europe	52.0
Japan	24.0
USA	24.0
Mean [Li +] µg/L [95% CI]	15.8 [7.05–24.6]
*Relation to suicide rate* (95% CI]	
Inverse correlation	32.0 [14.9–53.5]
Partial association*	44.0 [24.4–65.1]
No association	24.0 [9.36–45.1]

[*] Partial association includes reduced suicide risk selectively among men (9 studies) or women (2 studies), only at relatively low altitudes (2 studies), or only one period of time (1 study). Data are from recent reviews by Brown et al. (2018), Barjasteh-Askari et al. (2020), Memon et al. (2020), Araya et al. (2022), and Fadaei (2023), with references.

The available evidence suggests that lithium treatment may be even more effective than with anticonvulsants proposed as mood-stabilizers, or than antipsychotic drugs, including those with favorable effects in acute bipolar depression, although these comparisons require further study in adequately designed comparisons. Whether antidepressant treatments affect suicidal risk in bipolar disorder patients remains uncertain and their efficacy and safety for the treatment of bipolar depression has been controversial (Pacchiarotti et al. 2013). Nevertheless, the clinical association of suicidal risk with dysphoria, agitation, anger, aggression, and impulsivity strongly suggests that treatment with lithium or other mood-stabilizing medicines, perhaps including antipsychotic drugs, is likely to be safer and probably more effective than antidepressants in therapeutic efforts to reduce suicidal risk.

Methodological limitations of studies considered above include instances of reporting rates of suicidal acts that did not involve treated and untreated risk periods of similar length, or quite brief exposure times, especially in randomized-controlled trials, often with numbers of subjects that are disproportionately small relative to outcomes which are infrequent or even rare events such as suicidal acts (Wolf et al. 1996). Moreover, it is not clear how the timing of periods sampled might have influenced the results obtained. For example, some suicidal acts may be most likely to occur early in the course of bipolar illness among young patients (Jamison 1986; Tondo et al. 1998). In a recent nationwide Swedish study, the use of lithium as well as clozapine and ECT in young male patients (aged 15–19 years) were found significantly effective in preventing suicide (Desai-Boström et al. 2023). If effects of treatment on suicidal risk are age-dependent, as was found with antidepressants (Hammad et al. 2006; Barbui et al. 2009), an unrepresentatively low suicidal risk might arise during lithium treatment if this is long-delayed or if subjects are sampled at older ages.

Another limitation in many of the studies reviewed here is marked variation in the availability of potentially relevant clinical details, or inclusion of diagnostically heterogeneous cases, including some cases of schizoaffective disorder or recurrent unipolar depression, with varying suicidal risks. As a consequence, it is not always clear that patients given different treatments are closely matched by clinical details. Mirror-image studies (with versus without a treatment during matched periods of time) involve the same subjects and can confound comparisons by mismatching of the clinical status of patients at different times, particularly in a complex and often-changing illness like bipolar disorder. It is also possible that patients who accept, tolerate, benefit from, and continue to take lithium may not be clinically identical to those who refuse or discontinue such treatment and so may distort efforts at randomization.

Clearly, randomized and prospective trials involving explicit outcome measures relevant to suicidal risk are highly desirable, though difficult to carry out safely and effectively (Meltzer et al. 2003). Indeed, the clinical, ethical, and practical challenges of such trials are daunting and can benefit greatly from adequate support, as by corporations. Notably, however, commercial interest in the unpatentable natural product lithium, in particular, has always been low and decline of the use of lithium may well be associated with aggressive marketing of patent-protected medicines (Hogan 2017; Post 2018; Malhi 2023). Nevertheless, lithium has been included favorably in recent reviews about treatments aimed at reducing rates of suicide behavior (Lewitzka et al. 2013; Bastiampillai et al. 2017; Hogan 2017; Griffiths et al. 2023; Malhi et al. 2023) and recommended by the European Psychiatric Association (Wassermann et al. 2012).

Potential mechanisms by which lithium might reduce suicidal risk are not clear. They may involve preventing recurrences of high-risk mood-states (states with mixed manic-depressive features, agitation, dysphoria, depression) or represent a distinct action on suicidal and perhaps other aggressive or impulsive behaviors. Barraclough et al. (1972) and Coppen (1998) supported an association of antisuicidal effects with the mood-stabilizing actions of lithium. Others have found a correlation of mood-stabilizing and antisuicidal effects or have reported clinical observations of increased suicidal behavior after stopping lithium, independent of the previous responsiveness of mood symptoms to lithium therapy and suggested distinct antisuicidal or anti-aggressive actions of lithium (Müller-Oerlinghausen et al. 1992a, b; 2010 Lenz et al. 1994; Ahrens & Müller-Oerlinghausen 2001; Lewitzka et al. 2013; Del Matto et al. 2020). Another interesting hypothesis is that lithium may act in part to counter neurotoxic effects of other environmental chemicals such as lead (Brown et al. 2018).

Several findings suggest decreased physiological activity in serotonin-mediated neurotransmission in brain tissue of suicide victims, apart from effects of the means of suicide (such as poisoning and overdoses) that may confound the results. Increased abundance of serotonin 5-HT_2_ receptor sites found in postmortem brain tissue of suicides may reflect their up-regulation secondary to reduced presynaptic production or release of serotonin (Mann et al. 1986; Pandey et al. 2003; Savitz et al. 2009). Low concentrations of the main metabolite of serotonin, 5-hydroxyindoleacetic acid (5-HIAA), in cerebrospinal fluid of persons making violent suicide attempts also support a connection between deficient central serotonergic function and suicide (Moberg et al. 2011). In addition, Maes et al. (1995) found that seasonal variations in plasma concentrations of the serotonin precursor amino acid L-tryptophan correlated inversely with seasonal variations in the prevalence of violent suicide, complementing other findings of reduced tryptophan levels in depression. Finally, lithium treatment may tend to facilitate the production, release, and actions of serotonin in brain tissue (Müller-Oerlinghausen 1985; Price et al. 1990; Baldessarini 2013). Additional evidence supportive of that proposal may be arising from modern genomic research, including findings of associations between clinical responses to lithium treatment and genetic variants involved in regulating the production of the serotonin transporter protein (Tahroor et al. 2013).

Taken together, these observations may support the hypothesis that a decrease of cerebral serotonergic function may contribute to suicidal risk and might be countered selectively by lithium treatment (Kovacsics et al. 2009; Saunders and Hawton 2009; Müller-Oerlinghausen and Lewitzka 2010). However, the beneficial effect of increased central serotonergic function is at odds with the apparent lack of antisucidal effects associated with treatments with serotonergic antidepressants (Baldessarini 2013). It is likely that more complex effects on cerebral neurotransmission are involved, perhaps including a decrease of dopaminergic activity (Malhi et al. 2013) and a possible role of the glycogen synthase kinase-3β (GSK3β) enzyme since it modulates multiple systems that have been implicated in suicide and on which lithium exerts effects (Malhi et al. 2018).

## Conclusions

Findings summarized here, pertaining to research bearing on the hypothesis that lithium treatment may have a special role in reducing suicidal risk are encouraging, but it must be emphasized that such a role of lithium treatment is not securely demonstrated and has proved difficult to assess by use of randomized, controlled trials that support most clinically applied medicinal treatments. A major limitation of studies finding less suicidal risk during long-term treatment with lithium is that, to date, even in randomized controlled treatment trials, the outcomes involve incidental reporting of adverse events rather than testing for suicidal behavior as an explicit outcome.

An additional limitation—indeed, of all studies of therapeutic effects—is that patients who accept, tolerate, and adhere to long-term treatment with lithium may to some extent be self-selected and not entirely representative of the full spectrum of clinically encountered mood-disorder patients. Moreover, such factors as female sex, being married, having more education and employment, the presence of certain temperament or personality traits, relative improvement of depressive versus other symptoms with treatment, and the occurrence of adverse effects, as well as being less severely ill or ill for less time, all are likely to affect suicidal risk and so tend to confound interpretation of observed effects of treatment, especially in the absence of randomized studies with explicit outcome measures based on suicidal behavior.

Whether the same patients were assessed with and without lithium, or if subjects were randomly assigned to particular treatments in parallel groups, those who accept and continue the treatment may differ from others who fail to accept lithium treatment, tolerate it poorly, or discontinue early. Nevertheless, in order to test for long-term effects of *any* treatment, only patients who accept and tolerate it are available for analysis. Since a growing number of bipolar disorder and other mood disorder patients are reluctant to be treated with lithium, or to accept it for prolonged periods (Baldessarini 2013), it is increasingly important to identify alternative treatments that can reduce suicidal risk, particularly among medicines with commercial backing to support the challenging and expensive trials required to test for antisuicidal effects.

Ideal studies of such treatments would involve randomized assignment to specific and clinically plausible treatment options for prolonged periods and would involve primary outcome measures specific to suicidal behavior. Use of a placebo-control condition is unlikely to be feasible owing to ethical constraints when death is a possible outcome, and due to difficulties of recruiting patients into placebo-controlled treatment trials, especially over long times (Baldessarini 2013). Nevertheless, trials involving randomization to different but similarly plausible, even if unproved, treatments might be ethically and practically feasible, as in the InterSePT trial comparing clozapine with olanzapine in schizophrenia patients (Meltzer et al. 2003). So far, however, trials that compare, for example, lithium to an anticonvulsant or antipsychotic agent are rare and almost never continued for more than several months. Moreover, head-to-head comparisons of competing commercial products are unlikely to be favored by the pharmaceutical industry. It is important to have sufficiently large samples and exposure times as to be able to encounter substantial numbers of relevant though infrequent events of interest, particularly suicides and attempts. Studies yielding no such cases with any test treatment are uninterpretable and should be ignored.

Finally, lithium treatment requires closer monitoring of patients than with most other mood-stabilizing agents. This added level of clinical follow-up might facilitate identification of emerging symptoms associated with suicidal behavior, including suicidal ideation, and early agitation, dysphoria, anger, and insomnia or otherwise provide supportive and protective influences. We reported previously that various measures which can be considered indices of access to clinical care were closely correlated with state suicide rates in the United States (Tondo et al. 2006). However, the value of additional clinical contact and close supervision required by lithium treatment may not be a critical factor, given experience in the InterSePT trial for schizophrenia patients, which found superior antisuicidal effects of clozapine over olanzapine, despite matching for clinician contact-time (Meltzer et al. 2003). The proposed beneficial effect of lithium in reducing suicidal behavior should be considered as another reason to counteract the decline of use of this valuable agent that has emerged in recent years (Lewitzka et al. 2015; Post 2018; Malhi et al. 2023).

In conclusion, major mood disorders are associated with severe increases of suicidal behavior and fatalities, especially when the illnesses present as mixed, dysphoric-agitated states, sometimes accompanied by anger, aggression, or impulsivity. In such conditions, antidepressant treatments may lack a beneficial effect or even increase agitation and perhaps suicidal risk, whereas mood-stabilizers, and particularly lithium salts, maintained for prolonged times may be more effective treatments as a component of comprehensive clinical management aimed at suicide prevention.

## Data Availability

Not applicable.
